# Supplementary material for: Blocking Store-Operated Ca2+ Entry to Protect HL-1 Cardiomyocytes from Epirubicin-Induced Cardiotoxicity
Source: Cells. 2023 Feb 24;12(5):723. doi: 10.3390/cells12050723 (PMC10000558; doi:10.3390/cells12050723)
Supplement: Supplementary file 1 [file cells-12-00723-s001.zip › cells-2105001-supplementary.pdf]

Supplementary Materials

RNA-seq Data Analysis

Dataset: GSE217421 (https://www.ncbi.nlm.nih.gov/geo/query/acc.cgi?acc=GSE217421)

Human skin tissue, induced pluripotent stem cell, cardiac myocytes

Treatment condition: EPI (Epirubicin), 2uM for 48 hours.

The primary question is whether the listed genes are differentially expressed under Epirubicin (EPI) treatment compared to control (Table S1). Total 17 EPI samples and 56 control samples covering 5 different cell lines were used with each cell line having different number of replicates as shown in Table S2. Table S3 shows all the control cell lines and replicate numbers, except two not in the data set.

Table S1. Gene of interest comparison between EPI and control

Geneid	CTR	EPI	CTR-EPI	p-values	FC_EvsC
ATP2A1	6.3521	6.859	-0.5068	0.10356413	1.420923
ATP2A2	15.3132	15.0526	0.2606	0.177814885	0.83473
ATP2A3	7.8859	8.1625	-0.2766	0.449168353	1.211341
ITPR1	8.8424	8.3034	0.539	0.026119121	0.68823
ITPR2	8.4596	7.6078	0.8518	0.017024689	0.554093
ITPR3	7.5063	7.342	0.1643	0.42783548	0.892387
NFAT5	11.2428	9.9487	1.2941	1.88374E-08	0.40779
NFATC1	6.8247	6.4073	0.4174	0.137186378	0.748771
NFATC2	7.8592	6.886	0.9732	0.000154061	0.509376
NFATC3	10.1062	8.6265	1.4797	1.634E-10	0.358567
NFATC4	8.7542	9.1115	-0.3573	0.313684958	1.281017
NPPA	14.3383	16.431	-2.0927	2.92578E-07	4.265379
NPPB	12.5166	13.9646	-1.448	0.002760477	2.728341
ORAI1	8.0194	6.3349	1.6845	8.47074E-07	0.311114
ORAI2	9.436	9.0708	0.3651	0.081642536	0.776409
ORAI3	8.3587	7.2943	1.0645	2.33991E-07	0.478151
STIM1	10.2425	9.548	0.6945	6.82055E-05	0.61794
STIM2	8.6229	7.5348	1.0881	1.67131E-06	0.470379
TRPC1	8.9364	8.7972	0.1392	0.413543553	0.908042
TRPC2	0.233	2.4592	-2.2262	5E-13	4.679069
TRPC3	7.8435	7.0189	0.8246	9.81324E-05	0.564656
TRPC4	7.2956	4.8105	2.4852	6.3198E-09	0.178604
TRPC5	1.4794	0.6157	0.8638	0.016016663	0.549511
TRPC6	1.337	1.0743	0.2627	0.417846246	0.833533

Table S2. Table of trt by cell-final used						
trt	Cell Line					
	MSN01_03R_CM	MSN05_01R_CM	MSN06_07R_CM	MSN08_13R_CM	MSN09_04R_CM	Total
CTRL	11<br>15.07	12<br>16.44	11<br>15.07	12<br>16.44	10<br>13.70	56<br>76.71
EPI_	4<br>5.48	4<br>5.48	4<br>5.48	3<br>4.11	2<br>2.74	17<br>23.29
Total	15<br>20.55	16<br>21.92	15<br>20.55	15<br>20.55	12<br>16.44	73<br>100.00

Table S3. Table of State by Cell (all EPI and control samples)							
	Cell Line						
	MSN01-03R-CM	MSN02-04R-CM	MSN05-01R-CM	MSN06-07R-CM	MSN08-13R-CM	MSN09-04R-CM	Total
CTRL	12<br>15.00	5<br>6.25	12<br>15.00	11<br>13.75	12<br>15.00	11<br>13.75	63<br>78.75
EPI	4<br>5.00	0<br>0.00	4<br>5.00	4<br>5.00	3<br>3.75	2<br>2.50	17<br>21.25
Total	16<br>20.00	5<br>6.25	16<br>20.00	15<br>18.75	15<br>18.75	13<br>16.25	80<br>100.00

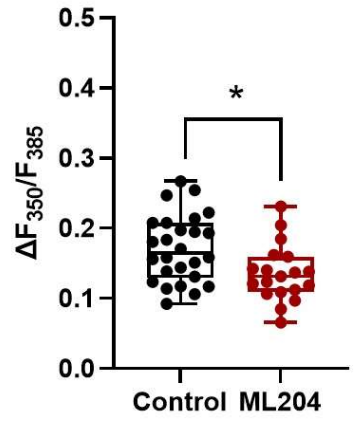

Figure S1. ML204 inhibited SOCE in HL-1 cells. HL-1 cells were treated with vehicle control or 10 μM ML204 for 30min. SOCE was measured following the same protocol in text after complete depletion of SR/ER Ca2+ stores by TG. Mean ± SD, *: $p<0.05$. (Based on t-test).

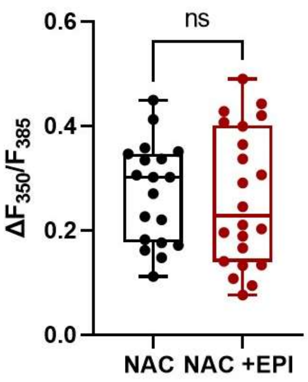

Figure S2. EPI failed to increase SOCE in HL-1 cells treated with ROS scavenger. HL-1 cells were incubated with 20 mM N-acetyl-l-cysteine (NAC), a ROS scavenger for 2 hours. Then the cells were either treated with vehicle control only (NAC group) or with 1μM EPI for 30min (NAC+EPI group). Mean ± SD, (based on t-test).

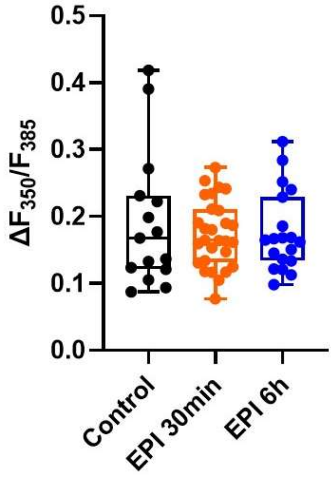

Figure S3. TG-sensitive SR/ER Ca2+ storage analysis in HL-1 cells. 10 μM TG induced intracellular Ca2+ elevation in HL-1 cells, reflecting SR/ER Ca2+ stores. Cells were treated with vehicle only (control), 1 μM EPI for 30min (EPI 30 min) or for 6h (EPI 6h). Mean ± SD, n>10. (based on One Way ANOVA). No significant differences among 3 groups.

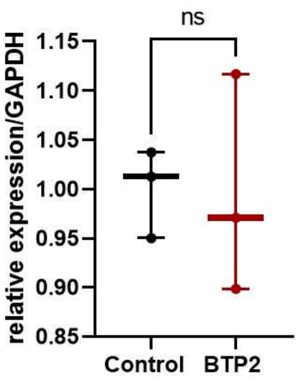

Figure S4. Orai1 expression is not affected by BTP2 treatment in HL-1 cells. Mean ± SD, n=3. (based on One Way ANOVA).
